# Supplementary material for: In Vitro and In Vivo Antibiofilm Activity of Red Onion Scales: An Agro-Food Waste
Source: Molecules. 2023 Jan 1;28(1):355. doi: 10.3390/molecules28010355 (PMC9822268; doi:10.3390/molecules28010355)
Supplement: Supplementary file 1 [file molecules-28-00355-s001.zip › molecules-2102312-supplementary.pdf]

In Vitro and In Vivo Antibiofilm Activity of Red Onion Scales: An Agro-Food Waste

Nermeen B. Ali [1], Riham A. El-Shiekh [1], Rehab M. Ashour [1], Sabah H. El-Gayed [1,2], Essam Abdel-Sattar [1,*] and Mariam Hassan [3,4]

[1] Department of Pharmacognosy, Faculty of Pharmacy, Cairo University, 11562 Cairo, Egypt
[2] Department of Pharmacognosy, Faculty of Pharmacy, 6th October University, 12585 Cairo, Egypt
[3] Department of Microbiology and Immunology, Faculty of Pharmacy, Cairo University, 11562 Cairo, Egypt
[4] Department of Microbiology and Immunology, Faculty of Pharmacy, Galala University, New Galala City, 43511 Suez, Egypt
* Correspondence: essam.abdelsattar@pharma.cu.edu.eg

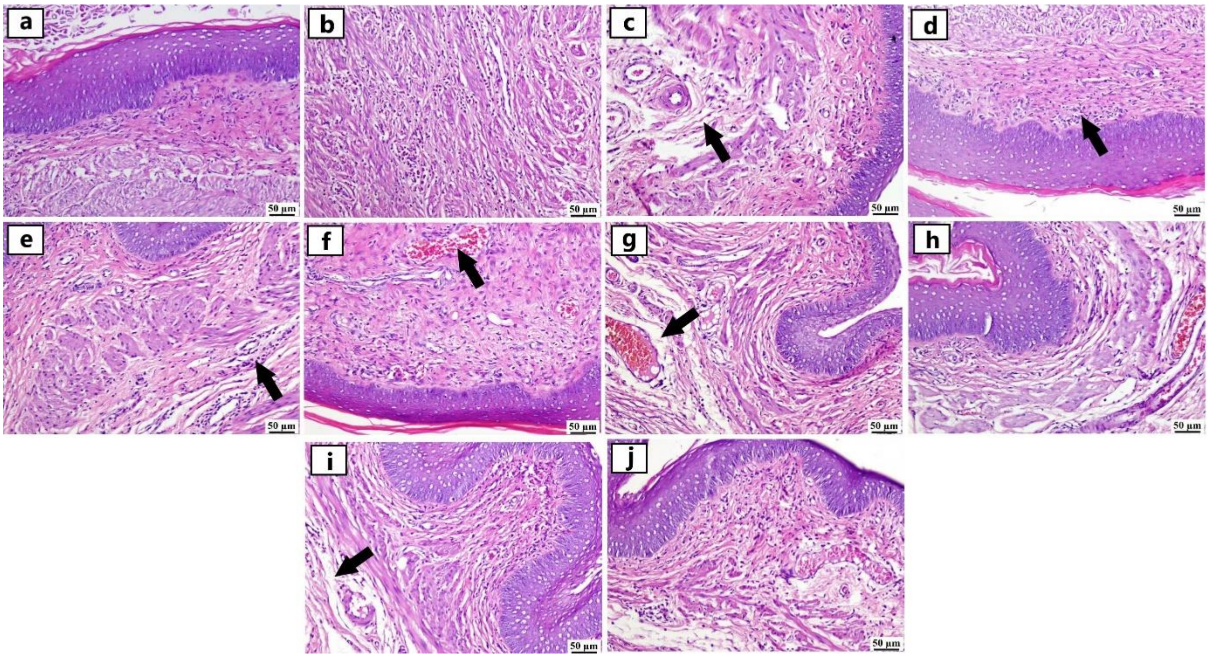

Figure S1. Photomicrograph of vagina sections of different experimental groups (H&E). (a) Gp1 showing normal stratified epithelium and propria of vaginal wall. (b) Gp2 showing inflammatory cells infiltration in lamina propria. (c) Gp3 showing mild edema in lamina propria with inflammatory cells infiltration (arrow). (d) Gp4 showing focal mononuclear inflammatory cells infiltration in lamina propria (arrow). (e) Gp5 showing moderate inflammatory cells infiltration in lamina propria (arrow). (f) Gp6 showing normal mucosa with few congested blood vessels (arrow). (g) Gp7 showing severely congested blood vessels with mild edema. (h) Gp8 showing mild edema. (i) Gp9 showing mild inflammatory edema in lamina propria (arrow). (j) Gp10 showing apparently normal mucosa.
